# White phosphorus munitions: pathophysiology, clinical management, and multidisciplinary perspectives on burn injuries and humanitarian challenges

**DOI:** 10.3389/fpubh.2025.1632840

**Published:** 2025-07-25

**Authors:** Mingchan Wang, Yaxing Bai, Xiaorui Zhang, Jing Zhang, Along Kang

**Affiliations:** ^1^Department of Pharmacy, Xijing 986 Hospital, Fourth Military Medical University, Xi’an, Shaanxi, China; ^2^Department of Dermatology, Xijing Hospital, Fourth Military Medical University, Xi’an, Shaanxi, China

**Keywords:** battlefield medicine, tactical medicine, white phosphorus munitions, chemical burns, battlefield first aid

## Abstract

White phosphorus (WP), a highly reactive and toxic substance, has been widely used in military applications. White phosphorus munitions (WPMs) embody a complex intersection of military utility and humanitarian concern, inflicting devastating injuries through their dual destructive mechanisms. These weapons induce severe thermal and chemical damage, penetrating deep into tissues to cause progressive necrosis and life-threatening systemic toxicity even with minimal exposure. This review synthesizes current understanding of WP injury pathophysiology—including hypocalcemia-induced arrhythmias, acute respiratory distress syndrome, and hepatorenal failure—while examining evidence-based interventions spanning battlefield first aid to advanced regenerative therapies. By integrating perspectives from military medicine, toxicology, and global health equity, this review provides a comprehensive foundation for clinicians, and researchers confronting the multidimensional challenges posed by WP injuries in conflict and post-conflict settings.

## 1 Introduction

White phosphorus munitions (WPMs), historically known for their significant destructive power, have played a complex and contentious role in warfare since their unique chemical properties were first discovered. First isolated through Hennig Brand’s serendipitous 1,669 experiment involving urine distillation, WP exhibits unique thermochemical properties—a low melting point (44.1°C), autoignition at 34–40°C under ambient conditions, and combustion temperatures exceeding 1,300°C (1–4). These attributes drove its rapid militarization, from British forces deploying WP grenades in World War I trenches to modern artillery shells used in asymmetric conflicts (5). Although Protocol III of the Convention on Certain Conventional Weapons (CCW) restricts WP use near civilians, its recurrent deployment in Fallujah, Gaza, Lebanon, and Ukraine (2022–2023) underscores persistent legal ambiguities (6).

However, the deployment of WPMs carries severe consequences, particularly concerning the profound harm they inflict on the human body. Upon ignition, these munitions produce extreme heat and toxic fumes, leading to extensive chemical burns and respiratory injuries. Such injuries cause significant pain, complicating medical treatment and extending recovery periods, often resulting in permanent disabilities or fatalities (7, 8). The pathobiology of WP injuries arises from synergistic thermal and chemical mechanisms. Combustion generates metastable P₄O₁₀ aerosols that hydrolyze into orthophosphoric acid (H₃PO₄) upon mucosal contact, inducing liquefactive necrosis. Crucially, lipid-soluble WP particles permeate fascial planes, causing delayed systemic toxicity through phosphide-induced Ca^2+^ chelation and mitochondrial dysfunction. Clinical reports confirm fatalities from ventricular arrhythmias even with <10% total body surface area (TBSA) burns, attributable to acute hypocalcemia (serum Ca^2+^ < 1.8 mmol/L) (9).

Current emergency protocols emphasize three imperatives: (1) immediate oxygen exclusion via immersion or wet dressings to halt combustion; (2) mechanical debridement over copper sulfate application due to nephrotoxicity risks; and (3) continuous cardiac monitoring for arrhythmia prevention. Emerging interventions like chelating hydrogels and extracorporeal phosphorus adsorption show preclinical promise but lack battlefield validation. Post-conflict environmental burdens further complicate recovery—unburned WP oxidizes into persistent phosphates that bioaccumulate in aquatic ecosystems, exemplified by Lebanon’s 2006 conflict where 462 hectares of farmland became non-arable (10).

Given these characteristics, the use of WPMs has attracted substantial international attention, leading to some regulatory restrictions. Despite these limitations, WPMs continues to be employed in certain regional conflicts, resulting in numerous civilian and military casualties. Consequently, raising awareness about WPMs burns and disseminating emergency response measures is of paramount importance. By synthesizing existing literature, we emphasize the severity of WPMs burns, enhance awareness of these specific injuries, and offer practical guidance for medical professionals and military personnel. Through this review, we hope to promote a deeper understanding of WP burns and provide direction and reference for future research and practice.

## 2 The physicochemical properties of WP

### 2.1 Chemical structure and stability

WP (chemical formula P₄) is one of the allotropes of phosphorus, typically appearing as a colorless, transparent, or slightly yellow crystalline solid (11). In its solid form, it is classified under the UN numbers 2,447, 1,381, and 1,338 (12). The presence of impurities can alter its color, resulting in what is known as yellow phosphorus. WP is extensively used in various industrial applications, including the production of semiconductors and fireworks, as well as in rodenticides and incendiary devices (12). It possesses a density of 1.82 g/cm^3^, a melting point of 44.1°C, and a boiling point of 280°C (13). Despite its significant industrial and military applications, WP is highly reactive and toxic, posing substantial risks to both the environment and human health.

The chemical structure of WP consists of a tetrahedron of four phosphorus atoms. Although this structure provides some stability, it places WP in a high-energy state, making it unstable at room temperature. It readily reacts with oxygen in the air to form phosphorus pentoxide (P₄O₁₀), releasing energy and demonstrating spontaneous combustion. This characteristic necessitates stringent safety measures during storage and use, typically requiring an airtight environment (14). Moreover, WP is nearly insoluble in water. Although water can extinguish burning WP, the compound can quickly reignite and produce smoke under dry conditions (13).

### 2.2 Combustion characteristics and reactivity of WP

WP is renowned for its distinctive combustion properties, which are central to its physical and chemical characteristics. With an exceptionally low ignition point of around 30°C, it is highly susceptible to spontaneous combustion at room temperature (7, 15–17). This susceptibility is further exacerbated by even minimal friction or impact, making its handling particularly hazardous (11). The combustion process of WP involves complex chemical reactions and significant physical effects, such as the emission of thermal and light radiation. When ignited, it produces a bright yellow flame accompanied by substantial heat and a dense cloud of white smoke. The temperatures generated during this process can reach approximately 1,300°C, far surpassing the typical 500–1,000°C observed in residential fires, thereby underscoring its potential danger (18). In addition to its high-temperature combustion, WP exhibits unique luminescent properties. In dark environments, it glows with a distinctive green light, a phenomenon known as chemiluminescence (19), resulting from its reaction with oxygen in the air. This reaction also produces a garlic-like odor and further white smoke (17). Beyond its reactivity with oxygen, WP is capable of vigorous reactions with various other chemicals, including halogens and sulfur. These reactions can lead to the formation of toxic byproducts such as phosphine (PH₃), which pose additional health and environmental risks (20). WP is highly lipophilic and can penetrate deep into tissues, leading to severe thermal and chemical burns (21). The high reactivity and potential hazards associated with WP necessitate stringent safety measures during its storage and handling. Typically, it is stored underwater or in inert atmospheres to prevent accidental ignition.

### 2.3 Molecular mechanisms of WP toxicity

**Figure 1:**
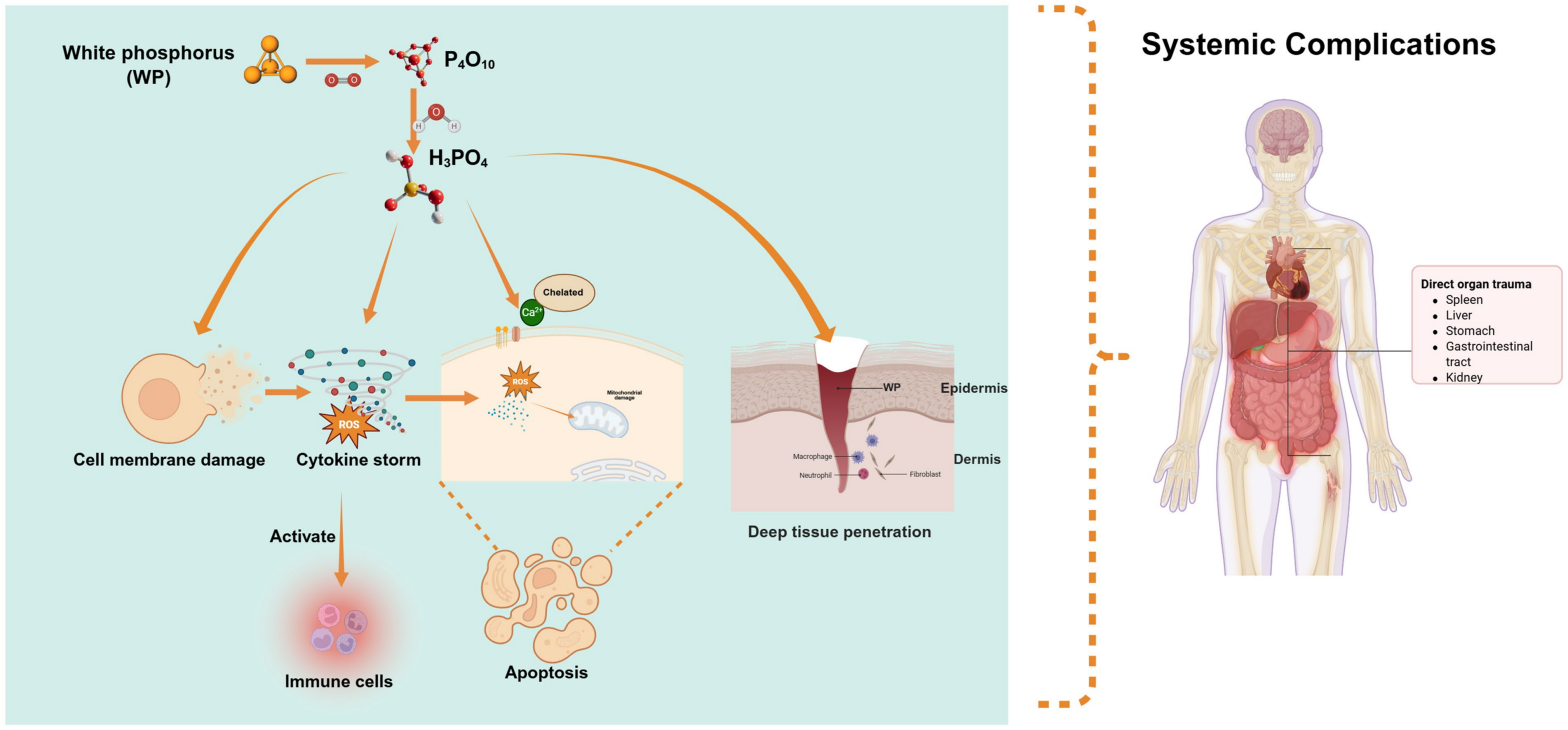
Molecular mechanisms of WP toxicity: the toxicity of white phosphorus stems from its combustion products (P₄O₁₀, H₃PO₄), which, via acidity and corrosivity, damage cell membranes, generate ROS, disrupt calcium homeostasis, and trigger intrinsic apoptotic pathways. Additionally, its lipophilicity enables deep penetration causing systemic toxicity (e.g., metabolic acidosis, multi-organ failure), while combustion products activate immune cells, exacerbate inflammation, and drive chronic complications.

WP is highly toxic, posing significant risks to human health (Figure 1). Classified as lethal by the Globally Harmonized System of Classification and Labeling of Chemicals (GHS) for both inhalation (H330) and ingestion (H300), its oral lethal dose (LD50) in humans is extremely low at 1.4 mg/kg (5, 22). Toxicity primarily stems from its combustion products—phosphorus pentoxide (P₄O₁₀) and phosphoric acid (H₃PO₄)—which exert multiple harmful effects at the molecular level (9).

These compounds directly damage cell membranes through their acidity and corrosivity, increasing permeability and triggering cell death, while releasing intracellular contents and activating inflammation. Simultaneously, the combustion process generates reactive oxygen species (ROS), causing oxidative stress that damages cellular components, impairs mitochondrial function, and induces apoptosis. Phosphoric acid also disrupts calcium homeostasis by binding to calcium channels, leading to abnormal signaling and cellular dysfunction. These combined effects—membrane damage, ROS generation, and calcium dysregulation—ultimately trigger intrinsic apoptosis pathways (7, 23–25). Moreover, WP’s lipophilicity enables deep tissue penetration and systemic toxicity, manifesting as metabolic acidosis, hypocalcemia, hyperphosphatemia, and multi-organ failure. Additionally, phosphoric acid and combustion products activate immune cells, fueling inflammation and contributing to chronic complications (4, 26, 27). Given its corrosive nature and far-reaching impacts, strict safety protocols are essential to prevent exposure and mitigate risks to both human health.

## 3 Symptoms of exposure to WP from explosive burns

WPMs, characterized by their pyrophoric properties and high chemical reactivity, inflict multifaceted trauma through combined thermal, chemical, and systemic toxic effects (28). Data from modern conflicts, such as the 2020 Nagorno-Karabakh war, reveal alarming injury patterns: 79.3% of victims sustained head injuries, 90.2% upper limb burns, and 46.3% lower limb involvement, with 37.9% exhibiting multiple shrapnel wounds. Notably, 28.7% required intensive care, and 10.3% succumbed to complications like acute respiratory distress syndrome (ARDS) and multi-organ failure within 30 days of hospitalization (29, 30).

### 3.1 Initial symptoms of exposure to WP

As the body’s largest organ, the skin is the first to bear the brunt when it comes into contact with WP. Upon contact, WP rapidly oxidizes at temperatures ranging from 34 to 40°C, generating extreme heat exceeding 800°C. This intense heat causes full-thickness burns, which are marked by yellow necrotic eschars and emit a pungent garlic-like odor (17, 31). The lipid-soluble P₄ molecules penetrate deep into subcutaneous tissues, continuing to cause progressive liquefactive necrosis even after the surface flames are extinguished.

Initial symptoms of WP burns include erythema, excruciating pain (more severe than sulfuric acid burns), and vesicular eruptions. In the extremities, these symptoms often progress to compartment syndrome. These burns are a combination of thermal and chemical injuries. The corrosive action of phosphoric acid, the heat generated by the reaction with phosphorus pentoxide, and the latter’s hygroscopic properties all contribute to extensive tissue damage (7, 13, 28). WP burns have a distinctive waxy yellow appearance in natural light and fluoresce under ultraviolet light. Compared to typical thermal burns, the healing process for WP burns is significantly slower. Full-thickness burns caused by WP are usually necrotic, necessitating immediate and effective medical treatment. If phosphorus particles are not completely removed from the burn site, they will keep reacting and generating heat until they are eliminated, the phosphorus is exhausted, or the oxygen supply is cut off (32).

In addition, the smoke produced by burning WP contains highly toxic and corrosive components like phosphorus pentoxide and phosphoric acid. Inhalation of this smoke irritates the respiratory system, triggering symptoms such as coughing, chest tightness, and breathing difficulties (33). Severe inhalation can lead to chemical pneumonia, pulmonary edema, or even respiratory failure. The irritants in the smoke also affect the eyes and mucous membranes, causing eye pain, tearing, and conjunctival congestion (34). Even in outdoor settings, low concentrations of WP smoke can immediately irritate the eyes, mucous membranes, and upper respiratory tract. In enclosed spaces, high concentrations can cause severe, irreversible damage to both the upper and lower respiratory tracts. WP particles can directly damage the cornea, leading to burns and potentially corneal perforation. Exposure to the smoke can also result in eye irritation, blepharospasm, photophobia, tearing, and conjunctivitis (35).

### 3.2 Subsequent symptoms of contamination by WP explosion

WP contamination from burns initiates a cascade of severe physiological disruptions upon entering the body. Inhalation can directly burn the lungs, heart, and other organs, and may also lead to systemic absorption (27, 28). Skin or mucous membrane exposure can similarly result in systemic uptake, triggering multi-organ dysfunction. This manifests as liver, kidney, and cardiac toxicity, alongside metabolic imbalances like hypocalcemia and elevated serum phosphorus levels. Blood abnormalities, including thrombocytopenia and coagulopathy, have also been observed. The central nervous system is affected, causing delirium, confusion, hallucinations, and coma. Moreover, the traumatic scene of a WP explosion—characterized by searing flames and acrid smoke—can inflict profound psychological trauma, often leading survivors to develop post-traumatic stress disorder (PTSD) with symptoms of persistent fear, anxiety, nightmares, and flashbacks (17, 23, 26, 36–38).

Systemic toxicity from WP unfolds in three distinct stages. The first stage is marked by gastrointestinal symptoms: upper abdominal pain, nausea, vomiting, loss of appetite, and occasionally jaundice (9, 20). These may be accompanied by headache, convulsions, coma, and cardiovascular failure; affected individuals’ breath, saliva, and vomit often carry a characteristic garlic-like odor. The second stage appears asymptomatic but shows early signs of toxic hepatitis upon liver histology examination (23, 24). By the third stage, occurring 4 to 8 days post-exposure, multi-organ failure sets in, encompassing neurotoxicity, bleeding tendencies, liver and kidney failure, and shock.

WP’s lipid solubility enables deep subcutaneous penetration, causing extensive necrosis. These burns are far more painful than typical thermal injuries and rapidly induce critical physiological changes. Within an hour of exposure, hypocalcemia, hyperphosphatemia, and calcium-phosphorus imbalances can occur (39, 40). Phosphoric acid, formed when phosphorus pentoxide reacts with water, consumes calcium (and potentially magnesium) during neutralization, forming insoluble calcium (magnesium) phosphate (19). This process depletes blood calcium and magnesium levels, with the degree of imbalance reflecting the amount of phosphorus “burned” in the body. In some patients, life-threatening hypocalcemia and hyperphosphatemia develop within the first hour, risking arrhythmias like QT interval prolongation, ST-T wave changes, bradycardia, or sudden death (9, 17, 26, 41).

The consequences extend beyond physical harm. Approximately 68% of survivors develop long-term neuropsychiatric disorders, including insomnia, hypervigilance, and PTSD, worsened by the weapon’s luminous smoke and recurring combustion events (29). Research by Khurshid et al. found that 42% of cases suffered memory impairment and executive dysfunction, likely due to lipid-soluble phosphorus metabolites crossing the blood–brain barrier. The psychological toll is compounded by disfigurement: 20.7% of victims sustain facial burns that impede social reintegration (32).

Environmental contamination further exacerbates the risk. In conflict zones, soil phosphorus levels can reach 14.1%, posing threats through residual particle ignition and groundwater contamination by phosphoric acid. This persistence turns acute burns into chronic wounds; 31% of cases develop Marjolin’s ulcers within 5 years. The ongoing cycle of harm underscores the need for integrated environmental decontamination in post-conflict medical strategies (42).

The complex interplay of immediate tissue damage, progressive systemic toxicity, and lasting psychosocial disability demands comprehensive, multidisciplinary approaches in conflict medicine.

## 4 Battlefield first aid for WP burns

WPMs impose catastrophic injuries through combined thermal-chemical damage and systemic toxicity. These characteristics mandate time-critical interventions to limit tissue destruction and systemic toxicity.

### 4.1 Core principles of emergency management

Effective battlefield care requires sequential implementation of five critical actions: (1) rapid removal of contaminated clothing to prevent secondary ignition; (2) oxygen deprivation through water immersion or moist dressings; (3) mechanical debridement of particles under low-light conditions; (4) continuous hypothermic irrigation (1–4°C) to suppress residual combustion; and (5) systemic monitoring for electrolyte abnormalities.

### 4.2 First aid for WP contamination in battlefield conditions

WP, a highly hazardous chemical with self-igniting properties in air, demands immediate isolation from oxygen sources upon exposure. The initial response includes prompt removal of all contaminated clothing and accessories to mitigate further injury (24, 43). Emergency Treatment Protocol is as follows:

#### 4.2.1 Oxygen deprivation and fire extinguishment

The first-line intervention involves extinguishing the burning WP by immersing the affected area in cold water (<25°C) or applying water-saturated dressings, which should be replaced every 5–7 min. Given its low melting point of 44°C, warm or hot water must be strictly avoided, as it can liquefy the phosphorus, increasing its surface contact and exacerbating tissue damage (28). Additionally, aggressive water flushing risks dispersing burning particles onto uninjured skin or rescuers, which may reignite upon drying (13).

#### 4.2.2 Particle decontamination

Mechanical removal of unburned phosphorus particles is a critical step. Visible particles should be carefully extracted using blunt forceps under the illumination of a Wood’s lamp, which causes WP to fluoresce. In the absence of a Wood’s lamp, Ultraviolet (UV) flashlights or fluorescent detection devices may substitute, leveraging WP’s fluorescent properties for particle localization (7, 31, 44). Gentle compression with moist gauze for 3–5 min can dislodge embedded particles from subcutaneous tissues (43, 45). Copper sulfate, traditionally used for particle visualization, is contraindicated due to its potential to cause glucose-6-phosphate dehydrogenase (G6PD) inhibition, leading to hemolytic anemia and hemoglobinuria. Instead, 1–3% silver nitrate solutions offer a safer alternative for identifying deeply embedded particles (29, 46).

#### 4.2.3 Wound management and stabilization

Following particle removal, continuous irrigation with cold saline is essential to ensure complete decontamination. High-pressure water jets, such as those from syringes, can dislodge tenacious embedded particles while maintaining a cold temperature to prevent phosphorus liquefaction. Before definitive treatment, cold saline-soaked gauze dressings provide interim protection against re-ignition. Pain control should be administered according to standard analgesic protocols (29, 47, 48).

#### 4.2.4 Transport and post-treatment care

Burned areas should be covered with moist, cool gauze to prevent re-exposure to air and subsequent reignition during transport. In cases where maintaining gauze moisture is challenging, hydrogel dressings like Water-Jel WJ110, composed of 96% water and containing antibacterial tea tree oil, offer effective cooling and infection prevention (49, 50). As alternatives, Moist Exposed Burn Ointments can be applied; in extreme emergencies (51), urine-soaked bandages may serve as a last-resort measure.

Immediately after injury, effervescent calcium and magnesium tablets should be administered both orally and topically (dissolved in water) to counteract potential hypocalcemia and hyperphosphatemia. In the subsequent 12 h, close monitoring of serum calcium and phosphorus levels, electrocardiogram changes, and signs of multi-organ failure is imperative, with assessments conducted hourly to detect and manage complications promptly (19).

### 4.3 Systemic treatment

Upon admission to the rear hospital’s emergency department, a Wood’s lamp examination is essential to identify and completely remove any residual WP on the skin. Post-particle removal, burn wounds require immediate cooling and cleansing. Cooling not only decelerates burn progression and alleviates pain but also minimizes deep-tissue damage. Rinsing with copious amounts of clean water is recommended, while avoiding forceful flows that may exacerbate wound injury. The cleansing process targets the elimination of remaining particles and contaminants, thereby reducing the risk of infection. To safeguard wound integrity, substances like soap and alcohol should be strictly avoided during cleaning (28).

Debridement, a cornerstone of treatment, involves excising necrotic and non-viable tissue. Immediate surgical debridement is often necessary and may need reiteration until all phosphorus particles are eradicated. Wounds should be inspected at least twice daily for new particles or areas of smoldering, which signal the need for further intervention. Covering the debrided area with a 5% sulfamylon solution between surgeries aids monitoring. Definitive wound closure should be deferred until thorough debridement is confirmed, at which point split-thickness skin grafting can proceed (29, 44, 50).

Medication and life-support measures are integral to first-aid management. Analgesics such as morphine or fentanyl are administered to control pain, while timely fluid and electrolyte replacement is crucial for preventing and treating shock, maintaining hemodynamic stability. In severe burns, antibiotics may be prescribed to combat infection, and corticosteroids can mitigate the inflammatory response (52, 53).

Clinicians must also assess the risk of systemic absorption in WP burn patients. Significant hypocalcemia can trigger arrhythmias, while inhalation of combustion smoke may lead to respiratory distress or renal failure. In such cases, fluid resuscitation combined with antibiotic therapy and dexamethasone administration may be warranted (33).

For critically ill patients, standard care includes appropriate fluid replacement and vigilant monitoring of electrolyte levels (especially calcium and phosphorus) and electrocardiograms (ECGs). These measures enable early detection and mitigation of complications like hypocalcemia, hyperphosphatemia, and arrhythmias (47). Fluid administration is titrated based on hourly urine output, aiming for 0.5–1 mL/kg/h (17, 54).

Topical treatments are key to burn management. Sulfamylon, a sulfonamide antibiotic, is commonly used for severe burns, serving as an adjunct for second- and third-degree injuries with efficacy against various Gram-positive and -negative bacteria, including *Pseudomonas aeruginosa* (52). Flaminal Forte, composed of a hydrous alginate polymer and a glucose oxidase–peroxidase bio-enzyme system stabilized by guaiacol, offers antibacterial action and continuous debridement (49, 55).

Innovative approaches enhance treatment efficacy. Pulsed lavage systems using 4°C saline reduce particle retention compared to static irrigation (56), While quantum dot-embedded dressings show potential in wound management, there is currently a lack of research on their ability to enable real-time phosphorus detection with a sensitivity of 0.1 μg/cm^2^, and further investigation is needed to explore their feasibility and practical applications in this regard (57).

Once the initial treatment phase concludes, patients transition to outpatient follow-up. This comprehensive care plan includes ongoing wound management, pain control, physical therapy, rehabilitation, and psychological support. Some may require skin grafting and interventions to prevent scarring and contractures (58). Emerging therapies like allogeneic mesenchymal stem cell transplantation show promise in accelerating wound healing (Table 1) (59, 60).

**Table 1:** Multiphase clinical management framework.

Phase	Time range	Treatment principle	Specific intervention	Detailed description
Acute phase	0–72 h	Implementation of the 3C Principle	Cooling	Maintain hypothermic irrigation at a temperature range of 1–4°C
			Coating	5% mafenide acetate-embedded dressings/water - gel WJ110
			Chelation	Initiate calcium supplementation when the serum Ca^2+^ level is less than 8.5 mg/dL, and conduct monitoring every 4 h
Subacute phase	72 h - 2 weeks	Metabolic Stabilization	Dynamic Electrolyte Monitoring	Perform dynamic electrolyte mapping using continuous biosensors
			Targeted Supplementation	Calculate the calcium deficit and implement the corresponding supplementation
			Multi-organ monitoring and Hepatic Detoxification Therapy	Administer exosome therapy derived from MSCs (61)
Rehabilitative phase	More than 2 weeks	Functional Restoration	Gradient Compression Therapy	Apply gradient compression therapy with a pressure of 30–40 mmHg (62)
			Scar Reduction Measure in Conjunction	Use silicone sheets eluting transforming growth factor-β3 (TGF-β3) (63)
			Neuromuscular Rehabilitation in Conjunction	Conduct neuromuscular electrical stimulation (64)

## 5 Conclusion

WPMs pose a significant and enduring threat in contemporary warfare. Their dual thermal and chemical action inflicts devastating multi-system injuries, inducing liquefactive tissue necrosis and systemic toxicity simultaneously. Despite international regulations under the Convention on Certain Conventional Weapons (CCW), definitional ambiguities—especially the exemptions for multipurpose munitions—enable their continued use in conflict zones.

The lipid solubility of WP allows it to penetrate deep into tissues and be absorbed systemically. Even minor burns (affecting ≤10% of the TBSA) can trigger fatal hypocalcemia and multi-organ failure. Prompt intervention is crucial. Immediate removal of particles, thorough saline irrigation, and application of hypoxia-inducing dressings like saline-soaked gauze can stop the combustion process. However, traditional treatments such as using copper sulfate are controversial due to the risks of hemolysis and nephrotoxicity. Systemic complications, including electrolyte imbalances (hypocalcemia, hyperphosphatemia) and coagulopathy, necessitate strict monitoring and calcium gluconate supplementation. Long-term management should also address multiple organ failure, psychological trauma, and socioeconomic reintegration for survivors.

To address these clinical and logistical challenges, future strategies should prioritize three critical advancements: first, advancing portable battlefield decontamination technologies to improve WP particle removal under resource-limited conditions; second, developing biodegradable alternatives (e.g., hexachloroethane) for smoke-screen applications to reduce reliance on harmful incendiary munitions; and third, integrating stem cell therapy and pH-neutral hydrogels into standardized battlefield protocols to enhance wound repair and mitigate systemic toxicity.

Notably, translating these strategies into practice requires robust evidence, yet conducting randomized controlled trials (RCTs) in conflict settings presents unique hurdles. These include ethical constraints related to enrolling combat casualties, variable environmental conditions, and limited access to standardized data collection infrastructure. Such barriers underscore the need for structured observational studies and multicenter registries to generate evidence for battlefield protocols—particularly for novel interventions like mesenchymal stem cell therapy.
