# Correction: White phosphorus munitions: pathophysiology, clinical management, and multidisciplinary perspectives on burn injuries and humanitarian challenges

**DOI:** 10.3389/fpubh.2025.1673762

**Published:** 2025-08-08

**Authors:** Mingchan Wang, Yaxing Bai, Xiaorui Zhang, Jing Zhang, Along Kang

**Affiliations:** ^1^Department of Pharmacy, Xijing 986 Hospital, Fourth Military Medical University, Xi'an, Shaanxi, China; ^2^Department of Dermatology, Xijing Hospital, Fourth Military Medical University, Xi'an, Shaanxi, China

**Keywords:** battlefield medicine, tactical medicine, white phosphorus munitions, chemical burns, battlefield first aid

In the published article, there was an error in affiliation 1. Instead of “Xijing Hospital Department, Fourth Military Medical University, Xi'an, Shaanxi, China,” it should be “Department of Pharmacy, Xijing 986 Hospital, Fourth Military Medical University, Xi'an, Shaanxi, China.”

The original article has been updated.

